# Evaluation of the Effect of Perfluorohexane Sulfonate on the Proliferation of Human Liver Cells

**DOI:** 10.3390/ijerph20196868

**Published:** 2023-09-30

**Authors:** Kyeong Hwa Sim, Hyeon Seo Oh, Chuhee Lee, Heesoo Eun, Youn Ju Lee

**Affiliations:** 1 Department of Pharmacology, School of Medicine, Daegu Catholic University, Daegu 42472, Republic of Korea; kyeonghwa3@cu.ac.kr; 2 Department of Neurology, Daegu Catholic University Medical Center, Daegu 42472, Republic of Korea; ohs0603@dcmc.co.kr; 3 Department of Biochemistry & Molecular Biology, School of Medicine, Yeungnam University, Daegu 42415, Republic of Korea; chlee2@ynu.ac.kr; 4 Research Center for Advanced Analysis, National Agriculture and Food Research Organization (NARO), Tsukuba 305-8604, Japan

**Keywords:** perfluorohexane sulfonate, human hepatocellular carcinoma, liver cancer, colony formation, cell-cycle progression

## Abstract

Perfluorohexane sulfonate (PFHxS) is a widely detected replacement for legacy long-chain perfluoroalkyl substances (PFAS) in the environment and human blood samples. Its potential toxicity led to its recent classification as a globally regulated persistent organic pollutant. Although animal studies have shown a positive association between PFHxS levels and hepatic steatosis and hepatocellular hypertrophy, the link with liver toxicity, including end-stage liver cancer, remains inconclusive. In this study, we examined the effects of PFHxS on the proliferation of Hep3B (human hepatocellular carcinoma) and SK-Hep1 (human liver sinusoidal endothelial cells). Cells were exposed to different PFHxS concentrations for 24–48 h to assess viability and 12–14 days to measure colony formation. The viability of both cell lines increased at PFHxS concentrations <200 μM, decreased at >400 μM, and was highest at 50 μM. Colony formation increased at <300 μM and decreased at 500 μM PFHxS. Consistent with the effect on cell proliferation, PFHxS increased the expression of proliferating cell nuclear antigen (PCNA) and cell-cycle molecules (CDK2, CDK4, cyclin E, and cyclin D1). In summary, PFHxS exhibited a biphasic effect on liver cell proliferation, promoting survival and proliferation at lower concentrations and being cytotoxic at higher concentrations. This suggests that PFHxS, especially at lower concentrations, might be associated with HCC development and progression.

## 1. Introduction

Since the accidental discovery of Teflon in 1938, the material has gained considerable attention in the chemical industries due to its exceptional chemical stability, water-repellency properties, and high surface activity [1]. The favorable properties of Teflon led to the development of closely related chemicals, namely, perluoroalkyl and polyfluoroalkyl substances (PFAS). Thereafter, PFAS-related products have been widely used in various applications including in textiles, food packaging, fire retardants, and other industrial applications [2,3,4].

PFAS have become ubiquitous in ecosystems and have been detected in humans [5] as well as in the environment and wildlife [6,7,8]. With the advancement of analytical and toxicological methods, various adverse effects of PFAS have been reported. In particular, eight-carbon congeners (C8), perfluoro-octane sulfonic acid (PFOS), and perfluorooctanoic acid (PFOA) are the most widely distributed and bio-accumulated, and they have been reported to be toxic. As a result of this probable toxicity, the production of these chemicals has been voluntarily discontinued by their manufacturer, 3M Company. Numerous substitutes for these legacy PFAS have been introduced, including perfluorobutanoic acid (PFBA), perfluorobutanesulfonic acid (PFBS), perfluorohexanoic acid (PFHxA), and perfluorohexanesulfonic acid (PFHxS), but these alternatives are also potentially toxic [9,10].

PFHxS, a six-carbon congener developed as a substitute for PFOS, is widely used in industrial and consumer applications, particularly in the electroplating industry [11], and is highly prevalent in the environment and living organisms. Due to its persistent, bio-accumulative, and toxic properties [12,13,14], PFHxS and its salts have been listed in Annex A of the 2021 Stockholm Convention on Persistent Organic Pollutants. Consequently, regulations on the use of PFHxS are being strengthened worldwide. For example, Korea and Japan are considering adding PFHxS to the PFAS survey items for water quality, which already includes PFOS and PFOA.

Studies on the toxico-kinetics of PFAS have shown that the distribution of PFHxS has a high liver-to-plasma ratio after oral and intravenous administration [15,16]. Animal studies have demonstrated that PFHxS promotes hepatic steatosis and exacerbates the hepatic symptoms of non-alcoholic fatty liver diseases (NAFLD) in obese mice [13,17]. Similarly, an epidemiological study has indicated a positive association between serum PFHxS levels and the NAFLD index in obese people [18], suggesting that PFHxS is potentially toxic to the liver. However, there is a scarcity of human studies specifically examining the effects of PFHxS on the liver, including the potential to cause liver cancer. Liver cancer was the third leading cause of cancer-related death worldwide in 2020 [19]. The identification of environmental factors contributing to the progression of liver cancer is crucial for developing effective therapeutic and preventative strategies for liver cancer. Hepatocellular carcinoma (HCC) is the most common form of liver cancer, accounting for 90% of cases, and is associated with poor prognosis [20]. Liver sinusoidal endothelial cells (LSEC) have been reported to play a role in hepatocellular carcinoma development and progression [21]. In this study, we investigated the effects of PFHxS on the proliferation of Hep3B (human HCC) and SK-Hep1 (human LSEC).

## 2. Materials and Methods

### 2.1. Materials

K^+^PFHxS (≥99.9% purity) was obtained from 3M Company (St. Paul, MN, USA). PFHxS was prepared by dissolving in dimethyl sulfoxide (DMSO). Antibodies of Cdk2, Cdk4, Cyclin D1, Cyclin E, PCNA, and GAPDH were purchased from Santa Cruz (Dallas, TX, USA). Anti-rabbit IgG and anti-mouse IgG were obtained from GeneTex (Irvine, CA, USA).

### 2.2. Cell Culture 

Hep3B and SK-Hep1 were obtained from the Korean Cell Line Bank (Seoul, Korea). Cells were cultured in minimum essential medium (MEM, GIBCO, Billings, MT, USA) supplemented with 10% fetal bovine serum (Hyclone, Logan, UT, USA) and 1% penicillin/streptomycin (GIBCO). The cells were maintained in a humidified atmosphere at 37 °C with 5% CO_2_.

### 2.3. Cell Viability Assay

Hep3B (8 × 10^3^/well) and SK-Hep1 (6.5 × 10^3^) cells were seeded in 96-well plates and cultured overnight, then cells with approximately 70% confluency were treated with various concentrations (0, 1, 10, 50, 100, 200, 300, 400, and 500 μM) of PFHxS for 24 or 48 h. Control cells were treated with vehicle control (DMSO) for 24 or 48 h. The plates were treated with tetrazolium salt 3-[4,5-dimethylthiazol-2-yl]-2,5-diphenyltetrazolium bromide (MTT) solution at 37 °C for 4 h. Formazan crystals were dissolved with DMSO, and the absorbance was measured at 595 nm as previously described [22].

### 2.4. Colony Formation Assay

Cells seeded into 24-well plates (4 × 10^4^ cells/well) were cultured overnight and then treated with different concentrations (0, 1, 10, 50, 100, 200, 300, 400, and 500 μM) of PFHxS for 12–14 days to allow colony formation. Colonies consisting of more than 50 cells were stained with 0.5% crystal violet (Junsei Chemical Co., Ltd., Tokyo, Japan) in 60% methanol. Images were acquired using the Chemi-Doc XRS imaging system (Bio-Rad, Hercules, CA, USA), and the number of colonies was quantified by measuring the optical density of the extracted crystal violet dye with DMSO at 570 nm.

### 2.5. Western Blotting

Cells lysates were prepared using lysis buffer containing 20 mM HEPES (pH 7.5), 1 mM EDTA, 1 mM EGTA, 10 mM NaF, 2 mM MgCl_2_, 150 mM NaCl, 10 mM KCl, 1 mM Na_3_VO_4_, 10 mM β-glycerophosphate, 1 mM DTT, 1 mM benzamide, 1 mM PMSF, 10 μg/mL aprotinin, 10 μg/mL leupeptin, 10 μg/mL pepstatin A, and 1% NP40. Equal amounts (20 μg) of proteins from each sample were separated using SDS-PAGE gel and were transferred onto a nitrocellulose membrane by Semi-Dry Transfer Cell (Bio-Rad, Hercules, CA) at 300 mA for 40 min. After blocking with non-fat dry milk, primary antibodies for CDK2 (1:1000), CDK4 (1:1000), Cyclin D1 (1:1000), Cyclin E (1:1000), PCNA (1:1000), and GAPDH (1:1000) were applied overnight at 4 °C, followed by incubation with secondary antibodies, anti-rabbit IgG (1:3000) and anti-mouse IgG (1:3000) for 1 h. Protein bands were detected using chemiluminescence reagents. Band intensities were analyzed using a Chemi-Doc XRS imaging system (Bio-Rad, Hercules, CA, USA). The membranes were reprobed with anti-GAPDH antibody, which was used as a loading control for each gel.

### 2.6. Statistical Analysis

Data are presented as mean ± SEM. Statistical analyses were performed using the two-sample *t*-test for two-group comparison or one-way ANOVA followed by Tukey’s post hoc test for multiple-group comparisons. A *p*-value less than 0.05 was considered significant. Statistical analysis was conducted using GraphPad Prism 4.0 software (GraphPad Software, Boston, MA, USA).

## 3. Results and Discussion

Exposure to PFAS potentially contributes to the development of NAFLD, which has become the most common cause of chronic liver diseases, including liver cancer, in recent years [23,24]. Furthermore, animal and human studies have demonstrated that exposure to PFHxS promotes NAFLD-related symptoms [17,18], raising the possibility that PFHxS may also affect the proliferation of liver cells.

**Figure 1:**
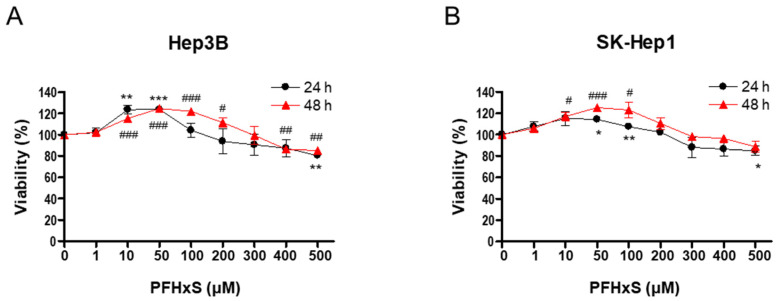
The effects of PFHxS on the viability of HCC and LSEC. Hep3B (**A**) and SK-Hep1 (**B**) cells were treated with the indicated concentrations of PFHxS for 24 and 48 h. Cell viability was measured using an MTT assay. Data represent the mean ± SEM (*n* = 3–4) (* *p* < 0.05, ** *p* < 0.01 and *** *p* < 0.001 vs. 24 h control; ^#^ *p* < 0.05, ^##^ *p* < 0.01 and ^###^ *p* < 0.001 vs. 48 h control).

To investigate the effect of PFHxS on the proliferation of HCC and LSEC, we assessed cell viability and colony formation in Hep3B and SK-Hep1 cells treated with different concentrations (0–500 μM) of PFHxS for 24 and 48 h. Treatment with PFHxS for 24 h significantly increased the viability of Hep3B cells at concentrations ≤ 50 μM and significantly increased the viability of SK-Hep1 cells at concentrations ≤ 100 μM. Similarly, treatment with PFHxS for 48 h significantly increased the viability of Hep3B cells at concentrations ≤ 200 μM and significantly increased the viability of SK-Hep1 cells at concentrations ≤ 100 μM. However, cell viability decreased compared with the control group in both cell lines at PFHxS concentrations ≥ 400 μM (Figure 1).

**Figure 2:**
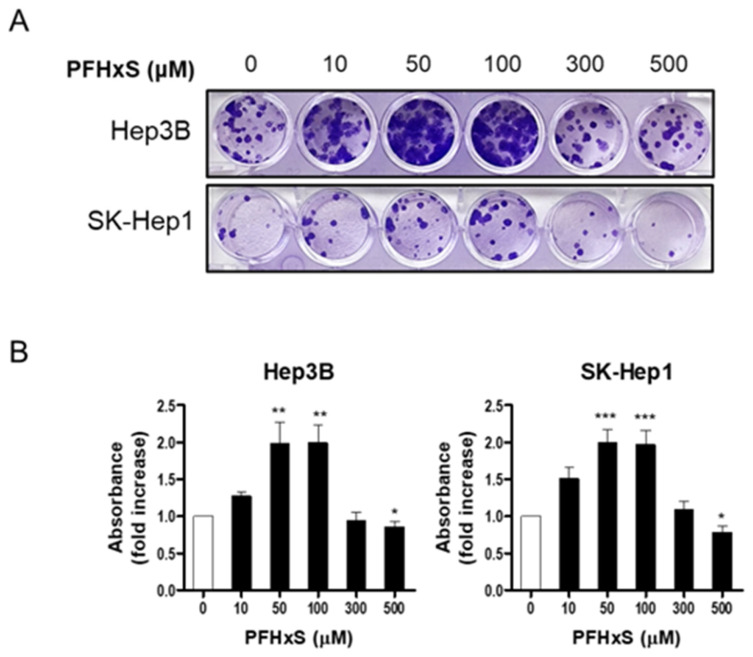
The effects of PFHxS on colony formation of HCC and LSEC. Hep3B and SK-Hep1 cells were treated with the indicated concentrations of PFHxS for 12–14 days. The colonies were stained with crystal violet and representative photos of the colonies are presented (**A**). The absorbance of resolved dyes was measured at 570 nm (**B**). Data represent the mean ± SEM (*n* = 6–12; * *p* < 0.05, ** *p* < 0.01 and *** *p* < 0.001 vs. control).

To confirm the stimulatory effect of PFHxS on liver cell proliferation, we measured colony formation. Cells were treated with PFHxS at various concentrations for 12–14 days. Consistent with the cell viability results, PFHxS significantly increased colony formation at concentrations ≤ 300 μM in both Hep3B and SK-Hep1 cells, while 500 μM of PFHxS decreased colony formation (Figure 2). These results indicate that PFHxS enhances liver cell proliferation at concentrations below 200 μM but becomes cytotoxic at concentrations above 400 μM.

**Figure 3:**
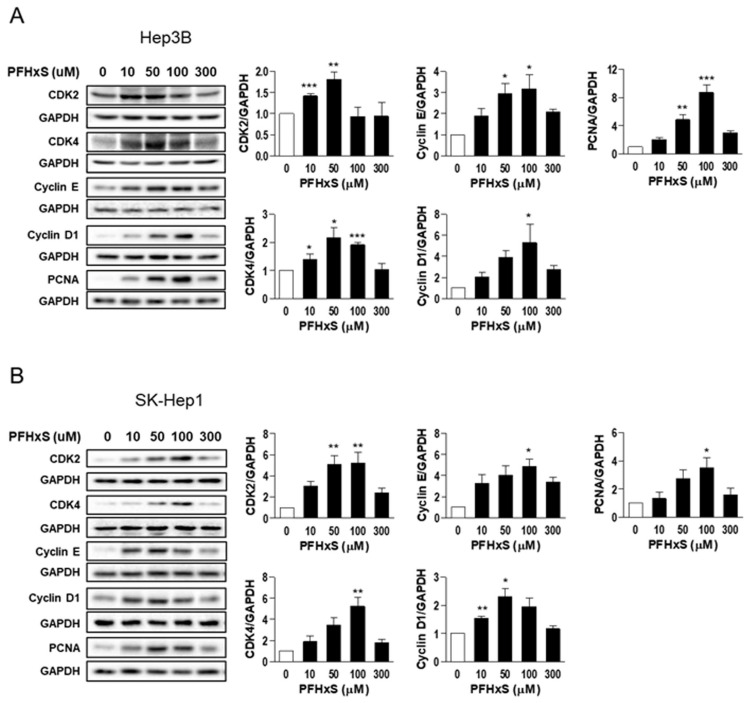
The effects of PFHxS on cell-cycle signaling molecules in HCC and LSEC. Hep3B (**A**) and SK-Hep1 (**B**) cells were treated with the indicated concentrations of PFHxS for 48 h. The protein levels of CDKs, cyclins, and PCNA were detected by Western blotting. The band intensities were measured and presented in the bar graphs. Data represent the mean ± SEM (*n* = 3) (* *p* < 0.05, ** *p* < 0.01 and *** *p* < 0.001 vs. control).

We also examined the effect of PFHxS on the levels of key regulators of the cell cycle, such as cyclins and cyclin-dependent kinases (CDKs), which drive cell-cycle progression from the resting G_0_ phase to growth phases, as well as the effect of PFHxS on proliferating cell nuclear antigen (PCNA), a marker of proliferation. Treatment of Hep3B and SK-Hep1 cells with 10–100 μM PFHxS for 48 h significantly increased the levels of cyclin E, cyclin D1, CDK2, CDK4, and PCNA (Figure 3).

Overall, our findings indicate that at concentrations below 200 μM, PFHxS stimulates liver cell proliferation by enhancing cell survival and colony formation. Conversely, at concentrations above 400 μM, PFHxS exhibits cytotoxic effects. The PFHxS-mediated increase in liver cell proliferation was associated with an increase in the level of cell-cycle markers. These observations align with previous studies on the cellular effects of PFOA and PFOS, which showed that the compounds increased cell viability at 100 μM but were cytotoxic at higher concentrations [25]. Recent epidemiological studies have also reported an association between elevated blood levels of PFOS and an increased risk of non-viral HCC and higher levels of alpha-fetoprotein, a hepatic tumor marker [26,27]. Although shorter-chain PFAS are generally considered to be less toxic than PFOA or PFOS, previous studies suggest that some shorter-chain replacements have similar or higher toxicity than long-chain PFAS, and the toxic potencies of PFAS cannot be determined by the chain length [28,29]. Olsen et al. (2007) reported that the serum concentrations of PFHxS in retired fluorochemical manufacturing ranged from 16 to 1295 ng/mL, with a half-life of approximately 4 years in the blood [30]. However, the specific concentrations of PFHxS in the human liver remain unclear. An animal study showed that oral gavage administration of PFHxS to rats (0–10 mg/kg/day) for 42 days resulted in a higher accumulation of PFHxS in the liver compared to serum levels. At a dose of 10 mg/kg, the ratio between liver and serum PFHxS concentrations was approximately threefold, and this ratio increased with both time and dose [12]. Therefore, it is plausible that liver concentrations in occupational workers may reach up to or more than 5 μM, a concentration relevant to our study, suggesting a potential impact of PFHxS on liver cancer cell proliferation. Future studies are needed to investigate the relationship between PFHxS in the blood and liver in humans.

In addition, multiple PFAS are detected in most environmental and biological samples. Recent studies have shown that PFAS mixtures can have synergistic, additive, or less than additive effects on liver cells, depending on the specific PFAS combination and the cellular targets [31,32,33]. Therefore, the potency of PFHxS in promoting HCC proliferation may depend on the exposure mode, such as exposure to PFHxS alone or in a mixture with other PFAS.

Although evidence regarding the initiation and carcinogenesis of liver cancer by PFHxS is lacking, our finding suggests that certain concentrations of PFHxS, a ubiquitous persistent organic pollutant, may exacerbate HCC progression. Considering that PFAS mixtures have been detected in human blood, the high distribution of PFHxS and PFOS in the liver, and the physiochemical similarities between PFOS and PFHxS, it is possible that PFHxS could increase the risk of HCC at concentrations lower than those identified in our study.

Our study provides insight into how a chemical found in the environment might contribute to HCC risk. However, larger-scale studies, including epidemiological and mechanistic investigations, are needed to validate these findings. Additionally, further research should explore the role of LSEC proliferation in PFHxS-increased HCC proliferation and how exposure to other individual substitute PFAS compounds, such as PFBS, PFBA, and PFHxA, as well as single or combined exposure to PFHxS and legacy PFAS, affects the development of HCC.

## 4. Conclusions

Our study suggests that PFHxS, a well-known alternative to PFOS, may contribute to the progression of HCC in humans. PFHxS increased the survival and proliferation of HCC and LSEC at relatively low concentrations (10–200 μM). Conversely, higher concentrations of PFHxS (>400 μM) were cytotoxic.

These findings provide valuable insights into PFHxS kinetics and facilitate the contextualization of human toxicity data, which is crucial for risk assessment of PFAS exposure in humans. In particular, this finding is of great importance in Japan and South Korea, where concerns about the potential toxicity of PFHxS are growing.

## Data Availability

The data presented in this study are available on request from the corresponding author. The data are not publicly available due the authors’ uncertainty about data sharing at the time the manuscript was submitted for publication.
